# Effect of frenotomy on breastfeeding and reflux: results from the BRIEF prospective longitudinal cohort study

**DOI:** 10.1007/s00784-020-03665-y

**Published:** 2020-12-14

**Authors:** Kirsten W. Slagter, Gerry M. Raghoebar, Inge Hamming, Jiska Meijer, Arjan Vissink

**Affiliations:** 1 Department of Oral and Maxillofacial Surgery, University Medical Centre Groningen, University of Groningen, Groningen, The Netherlands; 2 Imedi, Groningen, The Netherlands; 3 General Practitioners Research Institute, Groningen, The Netherlands

**Keywords:** Ankyloglossia, Lip-tie, Breastfeeding, Self-efficacy, Patient outcome assessment, Gastroesophageal reflux

## Abstract

**Objectives:**

To assess the Efficacy of Frenotomy with regard to Breastfeeding and Reflux Improvement (BRIEF) in infants with breastfeeding problems.

**Materials and methods:**

A cohort of 175 consecutive breastfeeding women with breastfeeding and reflux problems related to a tongue-tie or lip-tie fulfilling the inclusion criteria was longitudinally followed for 6 months. The effect of frenotomy on these problems was studied by a standardized oral assessment and completing the validated Breastfeeding Self-Efficacy Short Form (BSES-SF), nipple pain score (Visual Analogue Scale, VAS), and Infant Gastroesophageal Reflux Questionnaire Revised (I-GERQ-R) questionnaires pre-frenotomy and at 1 week, 1 month, and 6 months’ post frenotomy.

**Results:**

All 175 women completed the 1-month follow-up and 146 women the 6 months’ follow-up. Frenotomy resulted in a significant improvement of BSES-SF, nipple pain score, and I-GERQ-R after 1 week, which improvement maintained to be significant after 1 month for BSES-SF and I-GERQ-R, and after 6 months for I-GERQ-R. The improvements were irrespective of the type lip-tie or tongue-tie underlying the breast feeding and reflux problems. No post-operative complications were observed. About 60.7% of infants still was breastfed 6 months after treatment.

**Conclusions:**

Frenotomy is a safe procedure with no post-operative complications and resulting in significant improvement of breastfeeding self-efficacy, nipple pain, and gastro-oesophageal reflux problems.

**Clinical relevance:**

Frenotomy of a tongue-tie and or lip-tie can lead to improvement of breastfeeding and reflux problems irrespective of the type of tongue-tie or lip-tie and should be considered by clinicians as a proper tool to resolve these problems if non-interventional support did not help.

**International trial register:**

ISRCTN64428423

**Supplementary Information:**

The online version contains supplementary material available at 10.1007/s00784-020-03665-y.

## Introduction

Longitudinal studies have shown that mothers’ belief regarding their ability to breastfeed is one of the major predictors of prolonged exclusive breastfeeding [1]. Positive breastfeeding experiences strengthen breastfeeding self-efficacy [1] and have a positive impact on maternal wellbeing [2]. The World Health Organization considers breast milk as the best source of nourishment for infants. Exclusive breastfeeding is recommended up to 6 months of age [3], but worldwide only 40% of children under this age are exclusively breastfed [4]. Negative breastfeeding experiences force some mothers to stop earlier than desired [5].

There are many different causes for negative breastfeeding experiences such as poor weight gain, necessitating supplementation, poor latch, maternal nipple pain, and oral restrictions like a tongue-tie (ankyloglossia) and/or lip-tie. Ankyloglossia (either the decrease in mobility for the tongue by classic anterior tongue-tie or a submucosal restriction, a posterior tongue-tie) and a superior tethered labial frenulum can cause altered latch and sucking mechanics [6]. The suckling process is complex and multi-factorial [6]. A frenotomy of a tongue-tie (ankyloglossia) and/or tethered lip-tie when women experience pain during breastfeeding could be an option if non-interventional professional support does not help [7]. A frenotomy could, e.g., help women, who experience breastfeeding difficulties, to improve maternal functioning in early parenthood [8]. Studies [9, 10] show that a frenotomy, if adequately performed, can improve breastfeeding scores and relief nipple pain directly after intervention. Moreover, this procedure is safe with no serious complications. However, studies with longer follow-up after frenotomy are needed to study whether these effects are persistent.

Another factor associated with breastfeeding difficulties is gastroesophageal reflux. Gastroesophageal reflux is a common phenomenon in infants, but the differentiation between gastroesophageal reflux and gastroesophageal reflux disease can be difficult [11]. Symptoms of reflux are non-specific, and there is increasing evidence that the majority of symptoms may not be acid-related. In children with infant gastroesophageal reflux symptoms, clinical improvement has been suggested following a frenotomy of a tongue-tie [12].

The aim of the BRIEF study was to assess Breastfeeding and Reflux Improvement by the Efficacy of a Frenotomy in infants with breastfeeding problems up to 6 months after treatment. Breastfeeding self-efficacy for mothers was used as the primary outcome measure. Secondary outcome measures were nipple pain during breastfeeding, gastro-esophageal reflux symptoms, and complications up to 6 months after treatment.

## Materials and methods

### Study design

**Fig. 1:**
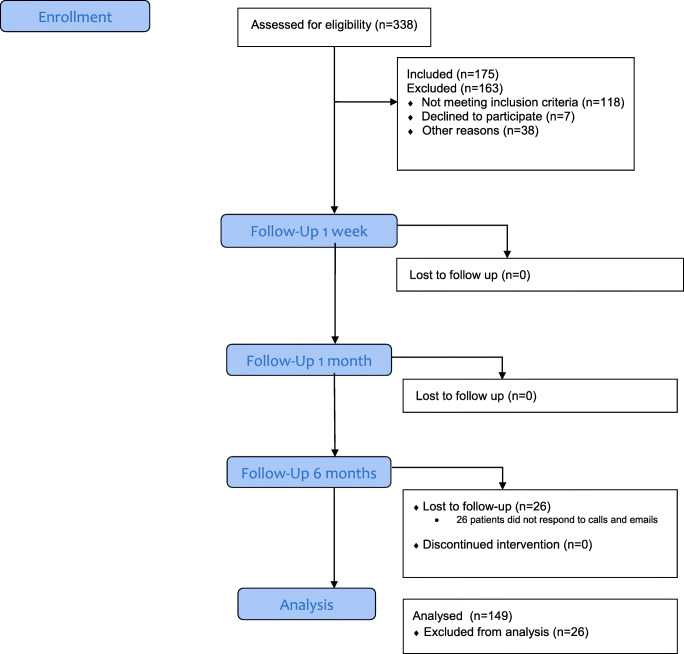
CONSORT flow diagram

The present prospective longitudinal cohort study was approved by the Medical Ethics Committee of the University Medical Centre Groningen, Groningen, The Netherlands (METc 2014/375), and registered in www.isrctn.com (ISRCTN64428423). Participants were 175 eligible consecutive breastfeeding women with healthy infants under 6 months with breastfeeding problems. The 175 eligible women were from a group of 338 women referred by external general practitioners or International Board Certified Lactation Consultants (IBCLC) to a private practice between October 2017 to April 2018 (Fig. 1). The other 163 mothers were not considered eligible for this study because their infants were prematures, twins, or were already revised for tethered maxillary labial frenulum (upper lip-tie) and/or ankyloglossia (*n* = 84), their infants received exclusively formula (*n* = 41), or their infants not seem to have oral restrictions (*n* = 38). Before entering the study, participants agreed and signed an informed consent.

### Oral assessment

All mothers that agreed to participate in the study had prior been seen by an IBCLC because of breastfeeding difficulties. A structured medical background history of mother and infant, pregnancy, birth, and breastfeeding history was completed by a doctor in dental surgery (K.S.) before the infants were orally assessed by the same doctor (K.S) and an IBCLC (I.H.). Mothers were assessed by the IBCLC (I.H.) for usual causes of breast or nipple pain such as nipple damage (abnormal latch/suck dynamic or breast pump trauma/misuse), dermatosis infection, and vasospasm [13].

**Fig. 2:**
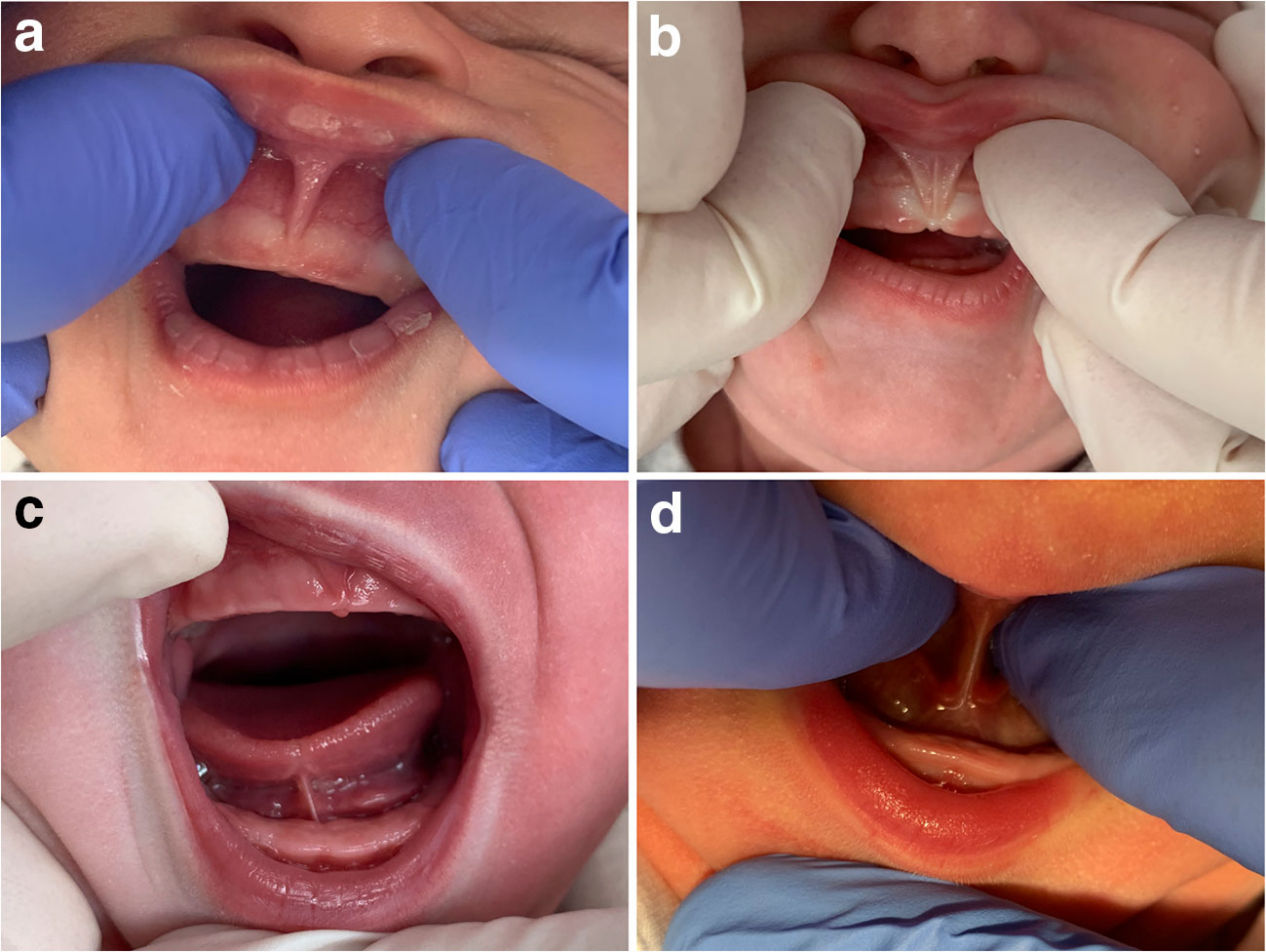
**a** Attachment in attached gingiva (Type 2). **b** Attachment in front of papilla (Type 3). **c** Attachment after tip to mid of the tongue (Type 2). **d** Attachment mid tongue to posterior (Type 3)

In order to perform a standardized oral assessment, a score form (supplementary file) was developed to record the different anatomical features using standardized classifications [7, 14] to describe frenula anatomy (Fig. 2). Further oral examination consisted of reporting sucking blisters, shape of the palate, retrognathia, location of attachment of the frenula, blanched frenula with elevation, anatomical restriction of elicited lateral lingual movement (impaired transverse tongue reflex), abnormal floor of mouth elevation of the tongue, and presence of thrush. The sucking evaluation consisted out of the notification of abnormal gum/lip pressure, cupping of the tongue against the finger, seal on the finger, and the nature of the sucking tongue movements.

### Treatment

A small amount of topical anaesthetic cream (xylocaine 5%) was applied with a cotton swab on the surgical site. The frenotomy procedure was performed by one doctor in dental surgery (K.S.) with electrosurgery (Servotome, Acteon Merignac France) using a sterilized tip. The dispersive electrode was placed under the patient. The tongue was elevated using a sterilized grooved director, while the tip of the active electrode was applied to the frenulum. Regarding (anterior) tongue-tie releases, midline tissue was incised starting at the anterior edge of the frenulum. An approximately 1-mm-deep central window was incised in the mucosa overlying the genioglossus muscle. The window in the mucosa was then extended laterally on both sides to release the mucosa, taking care not to disturb the fascia of the underlying genioglossus muscle. The appearance of a diamond-shaped wound was considered as a full release. Upper lip-tie releases were performed by lifting the upper lip, while the maxillary labial frenulum was released off the alveolar ridge up to the mucogingival junction. Immediately after the procedure, the infant was offered the breast or breastmilk by a bottle. Post procedural stretching exercises were advised to avoid reattachment of tissue by gently elevating the tongue and upper lip and massaging the wound four times per day for several weeks. Acetaminophen 60–120 mg suppository max 3 times per day was advised for analgesia if needed.

### Outcome measures and data collection

All study participants got access to the study-related outcome assessments prior to surgery, and at 1 week, 1 month, and 6 months after intervention via electronic correspondence using an Internet-based compliant survey portal (Typeform, Wordpress). All infants were followed clinically as per the office protocol. According to protocol, all patients had a routine follow-up after 1 week. When symptoms persisted or worsened following initial improvement, the mothers were offered a second procedure when a restriction was identified. During every follow-up visit, a routine assessment for post-operative complications was performed as described in a Cochrane review for tongue-tie [9]. Besides the study related outcomes, in addition, development in motor and cognitive growth after 6 months’ post-surgery was assessed [15]. Participating parents were asked to complete out questionnaires within 1 week by mail. Participants were excluded from the analysis if the 6 months’ questionnaires were missing.

### BSES-SF

Breastfeeding self-efficacy was measured using the validated Breastfeeding Self-Efficacy Short Form (BSES-SF) [16]. BSES-SF is a 14-item survey rated on a five-point Likert-type scale. The Likert scale ranged from 1 = “not at all confident” to 5 = “always confident.” Sum scores were calculated with a range from 14 to 70, with higher scores indicating higher levels of breastfeeding self-efficacy.

### VAS

To evaluate nipple pain with breastfeeding, the pain score was measured with the Visual Analogue Scale (VAS) [17] with a range from 0 to 10 with 0 = “no pain” to 10 = “severe pain.”

### I-GERQ-R

Infant gastroesophageal reflux was measured using the validated Infant Gastroesophageal Reflux Questionnaire Revised (I-GERQ-R) [18]. I-GERQ-R is a 13-item survey with strong internal consistency designed to evaluate the severity of gastroesophageal reflux symptomatology. The I-GERQ-R utilizes ordinal response scales to measure the severity of symptoms associated with infant gastroesophageal reflux disease (GERD). Scoring involves the summarization of 12 items (score range, 0–42), where lower scores reflect lower symptom severity.

### Statistical analysis

All data from the questionnaires and oral assessments were entered into an anonymized database by an independent research assistant (B.K.) Statistical analyses were performed by an independent statistician (C.P.) from the University of Groningen. Sample size estimations were determined using testing for differences in dependent mean values for breastfeeding outcome measures. Assuming a two-tailed test, an 0.05 alpha level, an 80% power, a conventional score of 56, and an equal standard deviation value of 10.5, a total of at least 152 subjects was required to detect a clinical difference on the mean BSES-SF (6 points difference in total score).

With regard to the analysis of the oral anatomy, an inter- and intra-examiner correlation analysis between the surgeon and the IBCLC was performed and repeated twice in a random order. A *p* ≤ 0.05 was considered to indicate statistical significance.

Distributions were verified for ordinal and continuous scale measures using graphical analyses. Differences in mean outcome measures between matched pairs of study time points were evaluated using Wilcoxon signed rank statistics. Regression analyses were performed to test for associations between the main outcome measures and physical characteristics. Study data were safeguarded by removal of protected health information and the assignment of unique study identification numbers for infants. A password-protected database was utilized. Statistical comparisons were analysed with (SPSS version 24; IBM Corp., Armonk, NY).

## Results

### Patient characteristics

The study sample consisted out of 175 eligible breastfeeding women with healthy infants out of 338 woman visiting the clinic during the study period. The characteristics of the study group are presented in Table 1. After 6 months, 146 patients were evaluable for outcome and the other 29 patients were lost to follow-up (Fig. 1) All patients but one received both a tongue-tie release and a frenotomy. Eight (4.6%) patients needed a second lingual frenotomy within 1 month after the initial treatment for either lack of improvement of symptoms or recurrence of symptoms after initial improvement.

**Table 1:** Characteristics of the breastfeeding woman who were eligible for this study

	Women
*n*	175
Age mother (years)	
Range	19–42
Mean (sd)	31.6 (3.9)
Age baby (*n*, %)	
0 months	90 (51.4)
1 month	49 (28)
2 months	24 (13.7)
3 months	12 (6.9)
Ethnicity (*n*, %)	
Dutch	171 (97.7)
Non-Western Immigrant	4 (2.3)
Education (*n*, %)	
University or college	130 (74.3)
Tertiary education	41 (23.4)
Secondary education	4 (2.3)
Pregnancy (*n*, %)	
Uncomplicated	162 (92.6)
Complications	13 (7.4)
Birth (*n*, %)	
Home	56 (32)
Hospital outpatient department	36 (20.6)
Hospital	83 (47.4)
Delivery (*n*, %)	
Caesarean section	14 (8)
Vaginal artificial	15 (8.6)
Vaginal	146 (83.4)
Boy/girl ratio	93:82
Child (*n*, %)	
First	78 (44.6)
Second	64 (36.6)
Third	27 (15.4)
Fourth or more	6 (3.4)

### Oral assessment

The pre-operative anatomical classification as scored with the standardized score form (supplementary file) and other oral clinical results are presented in Table 2. The anatomical assessments were repeated twice in a random order. The interobserver intraclass correlation coefficient was 0.83 (95CI 0.80–0.92). The intraobserver intraclass correlation coefficient was 0.88 (95CI 0.80–0.97) for observer one and 0.84 (95CI 0.80–0.95). During the pre-operative oral assessment, all different types of tongue-ties and lip-ties were seen (Table 2). Infants with lip-tie attachment in front of the anterior papilla (Type 3) or a tongue-tie attachment mid-tongue to posterior (Type 3) were most frequently seen. A large percentage of infants also had sucking blisters, high palate, and two coloured tongue pre-operative.

**Table 2:** Results of oral assessments (for illustrations of the various types of lip-ties, tongue-ties, sucking blisters, palate, and two coloured tongue see the score form in supplementary figure 1)

	Sample
*n*	175
Lip-tie (*n*, %)	
Minimal visible attachment (Type 1)	1 (0.5)
Attachment in attached gingiva (Type 2)	47 (26.9)
Attachment in front of anterior papilla (Type 3)	123 (70.3)
Attachment into hard palate or papilla area (Type 4)	4 (2.3)
Tongue-tie (*n*, %)	
Attachment complete to tip tongue (Type 1)	11 (6.3)
Attachment after tip to mid-tongue (Type 2)	58 (33.1)
Attachment mid-tongue to posterior (Type 3)	100 (57.1)
Attachment not visible only palpable (Type 4)	6 (3.4)
Sucking blisters (*n*, %)	
Yes	144 (82.3)
No	31 (17.7)
Palate (*n*, %)	
High	137 (78.3)
Flat	38 (21.7)
Two coloured tongue (*n*, %)	
Yes No	133 (76) 42 (24)

### Frenotomy efficacy

Frenotomy improved BSES-SF, I-GERQ-R, and VAS nipple pain scores significantly after 1 week (Table 3). This improvement was still significant 1 month after treatment for both BSES-SF and I-GERQ-R (Table 3). Six months after treatment, I-GERQ-R scores remained significantly better in the 49 infants that presented with gastro-oesophageal symptoms at baseline. More important, 60.7% of infants still received breastmilk 6 months after treatment (Table 3).

**Table 3:** Results of frenotomy

	Pre-operative	1 week post-operative	*P* value	1-month post-operative	*P* value	6-month post-operative	*P* value
Women (*n*)	175	175		175		145	
BFSE-SF (mean, sd)	44. (10.4)	48.2 (10.7)	< 0.001*	51.7 (10.2)	< 0.001*	52.1 (10.8)	0.642
VAS (mean, sd)	4.0 (2.7)	3.3 (2.1)	< 0.001*	3.2 (2.1)	0.895	3.6 (2.3)	0.187
Babies with reflux (*n*)	49	49		49		44	
I-QERQ-R (mean, sd)	21.8 (4.9)	17.5 (4.8)	< 0.001*	15.8 (5.8)	0.007*	13.6 (3.9)	0.001*
Breastfeeding (*n*, %)							
Only breastmilk	93 (53.1)	101 (57.7)		88 (50.3)		44 (30.3)	
Breastmilk by bottle	63 (36)	42 (24)		38 (21.7)		25 (17.2)	
Breastmilk and Formula	19 (10.9)	20 (11.4)		19 (10.9)		19 (13.1)	
Formula	–	12 (6.9)		30 (14.1)		57 (39.3)	

Subgroup interaction analyses were performed to investigate the potential confounding role of infant age, sex, and tongue/lip anatomic classification on outcomes. No significant correlations were found. This meant that the anatomical classification prior to surgery did not influence the outcome of the variables studied.

No post-operative complications were observed. In addition, motor and cognitive development was normal in all patients. In one (0.7%) patient there was temporary hyper granulated tissue of the wound. The majority of infants needed little, if any, analgesia post treatment.

## Discussion

To the best of our knowledge, this first study in the literature with a 6-month follow-up after a frenotomy [10] shows a positive effect on breastfeeding score, pain, and gastro-oesophageal reflux. A standardized treatment and follow-up protocol was applied to score both the baseline characteristics and effects of treatment in both the women and babies. Furthermore, post-operative complications of releasing the tongue-tie or lip-tie as well as complications in motor and cognitive growth after 6 months were not observed. This is in line with the literature [9, 10, 16].

Regarding the primary outcome measure, the BSEF-SF score, a significant improvement in the mean score after 1 week and 1 month post-operatively, was observed. Our results are in line with the results of other short-term prospective cohort studies on frenotomy [19, 20]. Moreover, this study shows that the early improvement of BSEF-SF scores persisted until 6-month post-operative. It seems that the treatment has a quick effect on low median BSEF-SF scores prior to treatment and that improvement of the BSEF-SF scores occurs fast after the frenotomy. Still the improvement in time seems similar among the infants, independently of the reported BSEF-SF scores before treatment.

The success of breastfeeding may contribute to a positive general sense of maternal self-efficacy [21]. Because breastfeeding is one of the first experiences in parenthood, it deserves attention as a potential source of maternal self-efficacy that can be observed among most new mothers [22]. Professionals within primary care should be aware of the impact that negative caregiving experiences, such as breastfeeding difficulties, may have on efficacy beliefs and may help women to establish new positive experiences to let them grow their self-efficacy. High breastfeeding self-efficacy significantly predicted increased maternal self-efficacy through the transition of parenthood and could be fully explained by a successful breastfeeding experience [23]. Women who had negative experiences, such as pain, had lower levels of breastfeeding self-efficacy compared with women who did not experience pain during breastfeeding [24]. Problems in the early phase of breastfeeding are a frequently cited reason to end breastfeeding prematurely [25]. Therefore, to increase the chance of success, further (lactation) support may contribute to self-efficacy beliefs [21].

Nipple pain is a major indicator of ankyloglossia and is often the driving force behind failure of breastfeeding [26]. Previous studies demonstrated that ankyloglossia can lead to unsuccessful breastfeeding and frenotomy leads to decrease in nipple pain [9, 10]. The hypothesis is that a frenotomy creates more space for the nipple in the mouth due to the release of oral restrictions. In our study nipple pain was a common initial complaint. A significant improvement of the VAS pain score was seen as early as 1 week post-operative. It seems that surgical treatment leads to a fast effect regarding nipple pain.

Gastroesophageal reflux is a common phenomenon in infants, but a differentiation between gastro-esophageal reflux and gastroesophageal reflux disease is difficult [11]. Symptoms are non-specific, and there is increasing evidence that the majority of symptoms may not be acid-related but air-related. Despite this, gastric acid inhibitors such as proton pump inhibitors are widely and increasingly used, often without objective evidence or investigations to guide treatment [27–29]. Several studies have shown that these medications are ineffective at treating symptoms associated with reflux in the absence of endoscopically proven oesophagitis. Decrease of gastro-esophageal reflux symptoms also decreases over time [11]. The correction of latch abnormalities caused by ankyloglossia indicates that the swallowing mechanism is related to aerophagia instead of acid. This study showed a significant clinical improvement [18] of reflux symptoms at all follow-up periods after frenotomy. One could speculate that the improvement of reflux symptoms might be related to the passing of time. However, the observation that the largest reduction of reflux symptoms was already seen 1 week post-operative suggests that the frenotomy was responsible for the decrease of reflux symptoms. As such, it can be hypothesised that lingual restrictions are associated with infant gastro-esophageal reflux symptoms via aerophagia. However, due to the complex and multifactorial nature of infant gastroesophageal reflux, and a lack of studies reporting on the correlation between oral restrictions and reflux symptoms, further investigation is warranted here.

One of the most clinical important findings is that 60.5% of the infants who presented with breastfeeding difficulties in this study had a combination of tethered lip-tie and posterior tongue tie (class III or IV ankyloglossia; see supplementary file). Due to the additional subgroup interaction analyses, it seemed that infants with posterior tongue-tie improved significantly after the intervention, similar to babies with a classic visible anterior tongue-tie. The implications of these findings are notable, as previously, the estimated incidence of ankyloglossia referred mainly to anterior ankyloglossia[9, 10].

Recently, a cadaver study showed that ankyloglossia and its surgical management would need revision due to the different anatomical structure in neonates compared with adults [30]. Diagnosing a posterior tongue-tie is not easy: it is only palpable posterior, but not visible. There is no consensus on treatment of this type of tongue-tie. In this study frenotomy caused the same improvement in infants with posterior tongue-tie as in infants with a classical anterior tongue-tie. The decision to include all types of tongue-tie in this study may represent a paradigm shift in our current understanding of frenotomy. Moreover, it represents a population of infants who could potentially benefit from this procedure. More importantly, it identifies a population of infants who may otherwise remain undiagnosed and untreated with breastfeeding difficulties as diagnosing the lingual restriction of the frenulum is not that easy. This strengthens the appearance of anterior and posterior attachment of the lingual tie and the importance of an experienced surgeon.

It is important to understand that maxillary labial restriction can also affect latch quality. A shortened labial frenulum prevents appropriate flanging of the upper lip. In addition, a common clinical observation following lip-tie release is that the baby can open the mouth wider, facilitating a deeper latch, although this improvement can also be seen in children who undergo lingual frenotomy without maxillary labial frenotomy. The attachment of the labial frenulum is typically at the gingival margin or on to the palate, comprising more than 93% of all normal labial frenula [31, 32]. Our study demonstrated that a low insertion of the labial frenulum is common and comparable with the literature [32]. The criteria used to determine if the lip was tethered and needed a frenotomy, therefore, is not the insertion point of the frenulum itself, but rather the presence of local restriction such as blanching of the frenulum with elevation, bony remodelling of the alveolar ridge, lip dimpling, and observed failure of lip flanging during nursing. Given the ubiquity of the presence and level of attachment in most infants, the anatomical appearance alone cannot be the reason for release of the superior labial frenulum. Therefore, during an oral assessment, it is also important to complete a comprehensive head and neck evaluation. Factors such as retrognathia and head abnormalities must be considered prior to proceeding with frenotomy.

The correct age for a frenotomy is a dilemma. A frenotomy “too early” risks criticism that the infant may still feed well without treatment, whereas treatment “too late” produces a worn-out mother and infant and raises the possibility that the baby may not breastfeed normally long term. Possibly, health care providers should be aiming for treatment when there are signs of breastfeeding difficulties and non-interventional procedures have not solved the problem within a few weeks. This study shows that both early and late interventions are effective.

This study has several limitations, most importantly the lack of a control-arm without intervention. A control group was not added because of ethical constraints as for most parents that were referred a frenotomy was felt as a last resort. Therefore, having no foresight on intervention was not considered an option. This is unfortunate as a control-arm would have provided an answer to the question whether frenotomy is the therapy of choice after non-interventional methods have failed. However, given the results, showing an immediate effect on critical outcome measures, the authors feel confident that this study adds to the data supporting the use of a frenotomy in patients that have exhausted all non-interventional options. Future studies should determine whether surgical treatment is better than non-interventional professional support only. Another limitation is the difficulty to assess which part of the procedure is most efficacious: a frenotomy or a lip-or tongue-tie. Given the known low risk of both procedures and the risk of suboptimal breastfeeding, we chose to optimise the anatomical situation regardless of which treatment options were needed; as shown before, most patients benefited from the combined procedure. Other studies described in the literature also encountered these difficulties [33, 34]. In other studies, controls who underwent a sham treatment frequently/mostly ended up in the intervention group [35–39]. A final study limitation that has to be mentioned is the overrepresentation of highly educated women in the current sample. However, women who initiate breastfeeding are usually higher educated compared with women who do not start with breastfeeding [40, 41], suggesting different pathways underlying breastfeeding decisions with for women with different backgrounds. Future studies should examine whether the findings of the current study concerning the relationship between breastfeeding and a frenotomy can be replicated in lower educated groups of breastfeeding women or how formula fed children react to a frenotomy.

The clinical challenge with mother and infants experiencing breastfeeding difficulties is to determine how to support mother and child in the most optimal way. The surgical release of a lingual frenulum is only one of many treatment or support options. For example, in some infants’ interventions such as adjusting the mother’s positioning and attachment technique may resolve breastfeeding difficulties. More accurately determining which infant will derive breastfeeding benefit from a frenotomy will help avoid unnecessary surgery and prevent delays in accessing the most appropriate breastfeeding support. Clinically, a standardised assessment of frenulum function and anatomy is required, along with early breastfeeding assessment and support by either a lactation consultant or midwife with additional training, before tongue-tie or lip-tie release surgery is performed. A frenotomy should not be considered as a quick surgical solution for more complex breastfeeding problems. Moreover, when surgically intervening with lip- or tongue-tie-related breastfeeding difficulties, monitoring and lactation support should always be offered post-operative.

## Conclusion

Frenotomy of a tongue-tie and or lip-tie is a safe procedure with no reported post-operative complications after 6 months, if done by an experienced surgeon. Surgical release of the tethered oral tissues was shown to result in significant improvement of breastfeeding self-efficacy, nipple pain, and gastroesophageal reflux problems. Improvements occur early (1 week postoperative) and continue to improve to 6-months postoperative. Improvements were demonstrated in both infants with classic anterior tongue-tie and posterior tongue-ties. Based on this finding, clinicians should mark posterior tongue-tie as a potential aetiology of breastfeeding difficulties. The importance of clinical judgement and determining whether the cause of the breastfeeding difficulty can be surgically treated should be taken after non-interventional professional support did not help and before the surgical treatment takes place.

## Supplementary Information

ESM 1 (PDF 2340 kb)
