# Supplementary material for: Effect of frenotomy on breastfeeding and reflux: results from the BRIEF prospective longitudinal cohort study
Source: Clin Oral Investig. 2020 Dec 14;25(6):3431–9. doi: 10.1007/s00784-020-03665-y (PMC8137608; doi:10.1007/s00784-020-03665-y)
Supplement: Supplementary file 1 — (PDF 2340 kb) [file 784_2020_3665_MOESM1_ESM.pdf]

BOEFjes studie

Scoreformulier

Scoreformulier behorende bij het BOEFjes onderzoek (BorstvoedingsOnderzoek Effect Frenulotomie). Op dit formulier kan de arts of lactatiekundige vastleggen welk type tongriempje en/of lipbandje gezien is. De resultaten worden gebruikt voor een onder-zoek naar de effecten van frenulotomie op borstvoedingsproblemen en refluxklachten.

Graag aankruisen wat uw bevindingen zijn s.v.p. Graag één antwoord per vraag aankruisen

Naam baby:

Geboortedatum:

Behandeldatum:

Onderzoeksnummer:

BOEFjes studie

Scoreformulier

Welk type lipbandje ziet u?

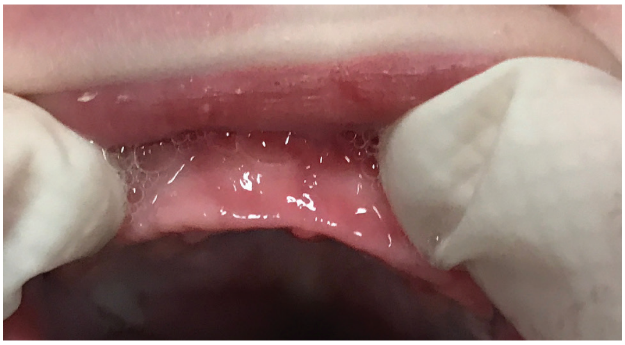

☐ **Type 1**

Bij heffen van de bovenlip tot maximale positie is het lipbandje amper zichtbaar. Beweeglijkheid is niet beperkt. Normale situatie.

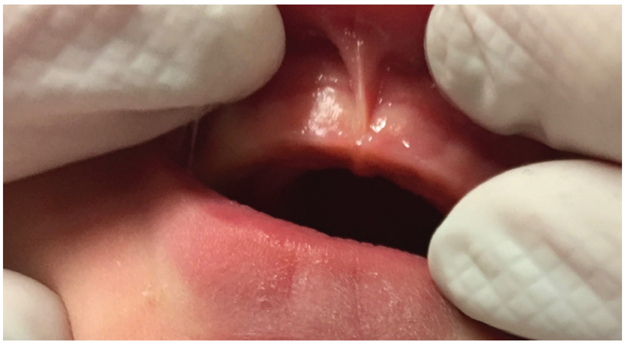

☐ **Type 2**

Bij heffen van de bovenlip tot maximale positie is zichtbaar dat het lipbandje doorloopt tot halverwege de bovenkaak.

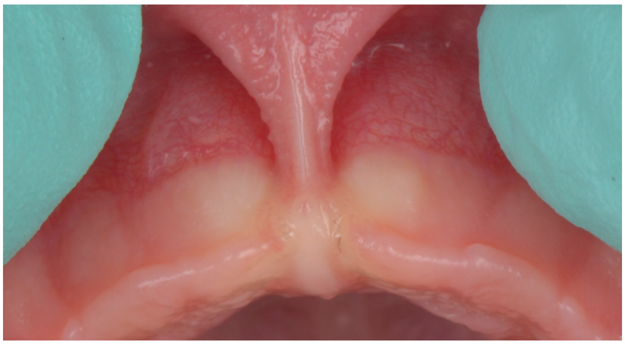

☐ **Type 3**

Bij heffen van de bovenlip tot maximale positie is zichtbaar dat het lipbandje doorloopt tot aan de rand van de bovenkaak.

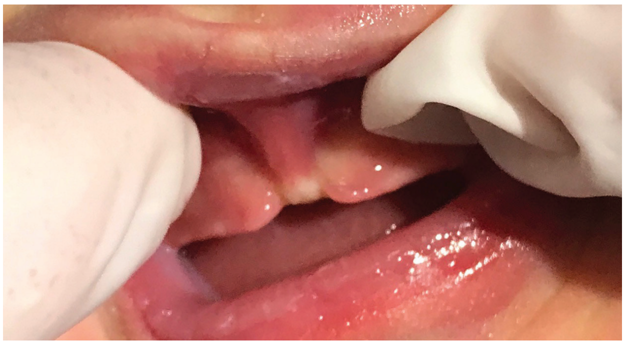

☐ **Type 4**

Bij heffen van de bovenlip tot maximale positie is zichtbaar dat het lipbandje doorloopt tot over de rand van bovenkaak, tot op het harde verhemelte.

BOEFjes studie

Scoreformulier

Welk tongriempje ziet u?

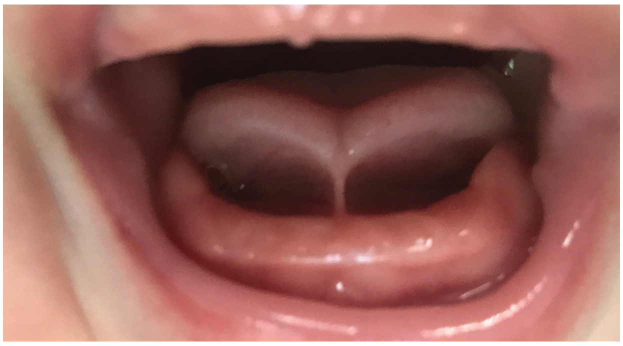

☐ **Type 1**

Duidelijk tongriempje zichtbaar onder de tong. Dit loopt door tot aan de punt van de tong. Bij uitsteken wordt punt van de tong hartvormig. Tong kan tevens posterior vastzitten.

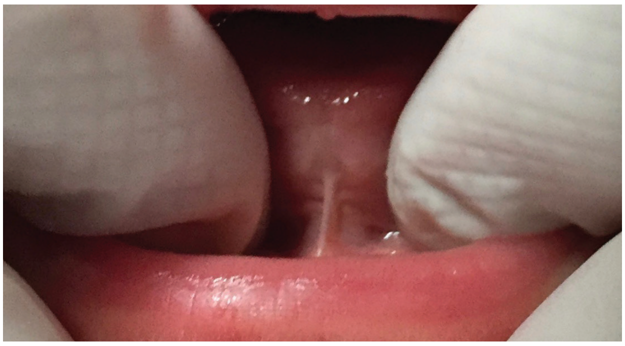

☐ **Type 2**

Duidelijk tongriempje zichtbaar onder de tong. Dit hecht halverwege de tong aan. Bij uitsteken wordt de tong beperkt en is hartvormig of vierkant. Tong kan tevens posterior vastzitten.

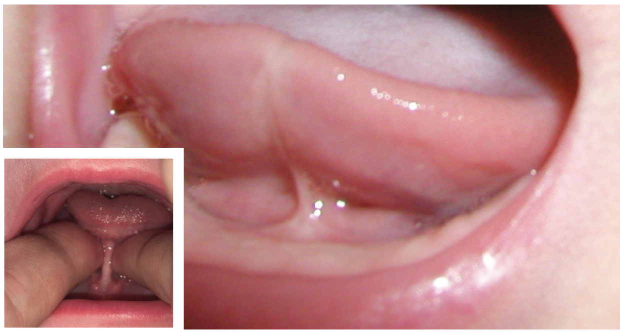

☐ **Type 3**

In de diepte is nog net een tongriem zichtbaar. Bij de Murphy maneuver is een duidelijke weerstand voelbaar, als hekje of snaar. De voor- en zijkant van de tong gaan omhoog, maar het midden zit vast.

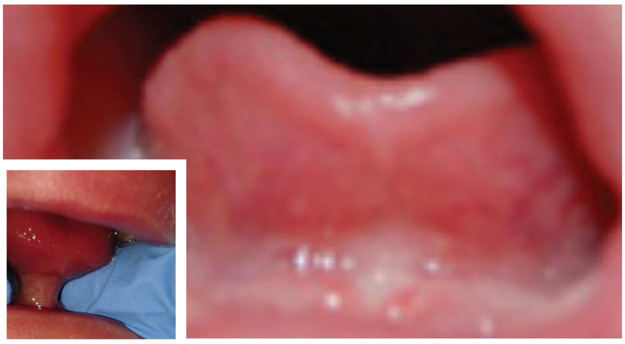

☐ **Type 4**

Tongriempje is niet direct zichtbaar, zit achter het mondslijmvlies verborgen. Bij optillen van de tong met vinger of sleufsonde wel zichtbaar. Bij de Murphy maneuver is een duidelijke weerstand voelbaar, als hekje/snaar of verkeersdrempel/ boomstam. Voor- en zijkant van de tong gaan omhoog, het midden zit vast.

BOEFjes studie

Scoreformulier

Ziet u zuigblaren?
Uitleg vraag

☐ Ja ☐ Nee ☐ n.v.t

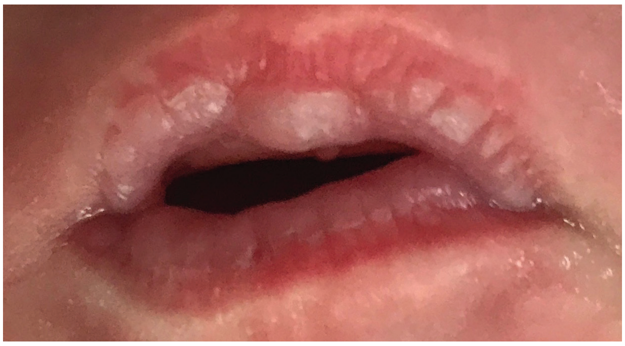

Is het gehemelte hoog?
Uitleg vraag

☐ Ja ☐ Nee ☐ n.v.t

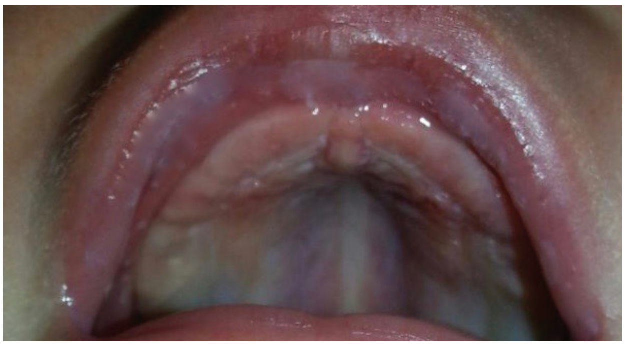

Murphy maneuver
Bij duwen op de plek van het frenulum deukt de tong bovenop in. Er is een kuiltje zichtbaar midden op de tong.

☐ Ja ☐ Nee

Bij het bewegen van een vinger over de mondbodem van de ene naar de andere kant voelt u:

☐ Weerstand ☐ Geen weerstand

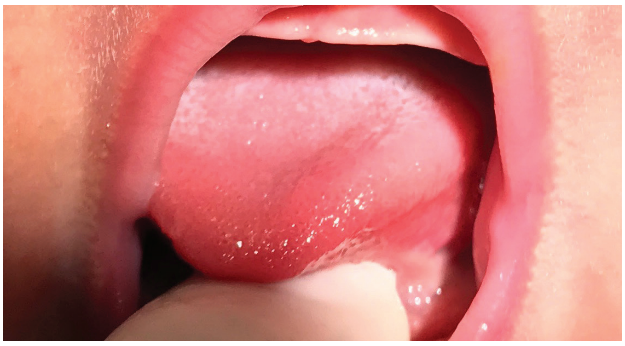

Zit de tongriem aan twee zijden vast?
Vast aan de binnenzijde kaak en aan de tong (eifeltoren vorm).

☐ Ja ☐ Nee

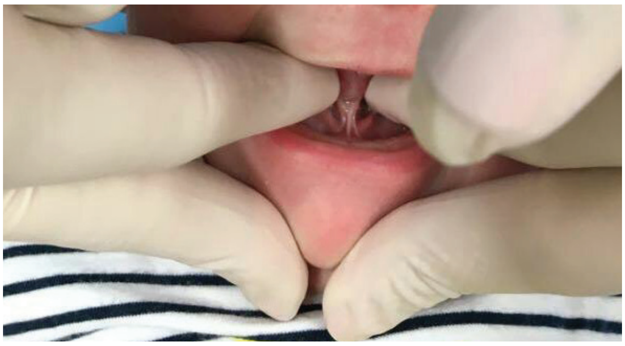

BOEFjes studie

Scoreformulier

Ziet u 2 kleuren op de tong?

Is er verschil waarneembaar tussen het voorste en achterste gedeelte van de tong mbt een witte aanslag?

☐ Ja ☐ Nee

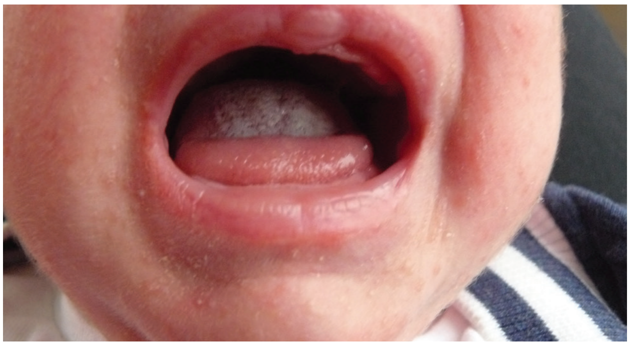

Welke tong beweging heeft uw kind?

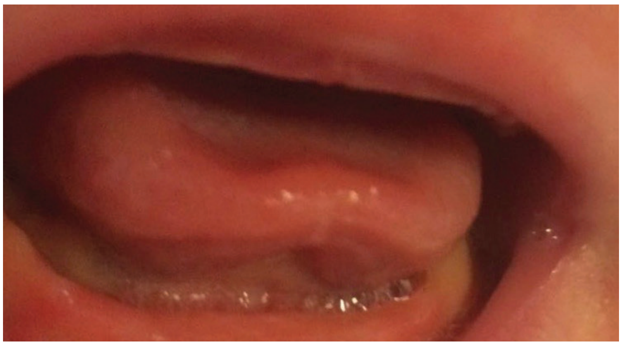

☐ **Alleen anterior**

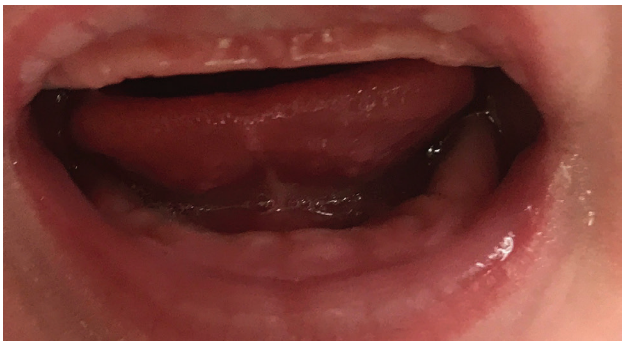

☐ **Midden en anterior**

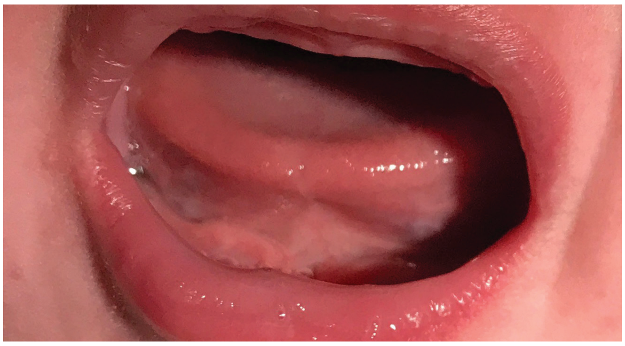

☐ **Zijranden (komvorm)**

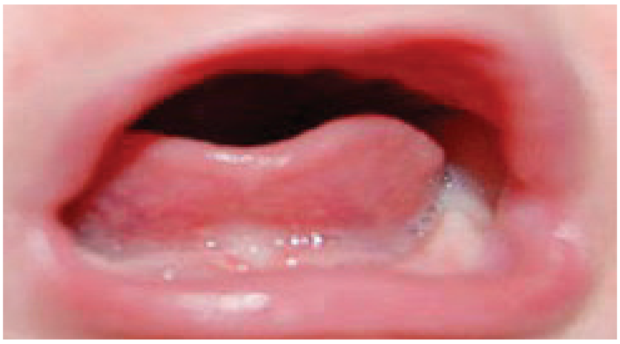

☐ **Posterior**

BOEFjes studie

BorstvoedingsOnderzoek Effect Frenulotomie
